# Circulating and Tumor-Infiltrating NK Cells From Clear Cell Renal Cell Carcinoma Patients Exhibit a Predominantly Inhibitory Phenotype Characterized by Overexpression of CD85j, CD45, CD48 and PD-1

**DOI:** 10.3389/fimmu.2021.681615

**Published:** 2021-06-04

**Authors:** Andrea Ziblat, Ximena Lucía Raffo Iraolagoitia, Sol Yanel Nuñez, Nicolás Ignacio Torres, Florencia Secchiari, Jessica Mariel Sierra, Raúl Germán Spallanzani, Agustín Rovegno, Fernando Pablo Secin, Mercedes Beatriz Fuertes, Carolina Inés Domaica, Norberto Walter Zwirner

**Affiliations:** ^1^Laboratorio de Fisiopatología de la Inmunidad Innata, Instituto de Biología y Medicina Experimental (IBYME-CONICET), Buenos Aires, Argentina; ^2^Centro de Educación Médica e Investigaciones Clínicas “Norberto Quirno” (CEMIC), Servicio de Urología, Buenos Aires, Argentina; ^3^Departamento de Química Biológica, Facultad de Ciencias Exactas y Naturales, Universidad de Buenos Aires, Buenos Aires, Argentina

**Keywords:** NK cells, renal cell carcinoma, CD85j, CD45, CD48, PD-1

## Abstract

Although natural killer (NK) cells infiltrate clear cell renal cell carcinomas (ccRCC), the most frequent malignancy of the kidney, tumor progression suggests that they become dysfunctional. As ccRCC-driven subversion of NK cell effector functions is usually accompanied by phenotypic changes, analysis of such alterations might lead to the identification of novel biomarkers and/or targets in immuno-oncology. Consequently, we performed a phenotypic analysis of peripheral blood NK cells (PBNK) and tumor-infiltrating NK cells (TINK) from ccRCC patients. Compared to HD, PBNK from ccRCC patients exhibited features of activated cells as shown by CD25, CD69 and CD62L expression. They also displayed increased expression of DNAM-1, CD48, CD45, MHC-I, reduced expression of NKG2D, and higher frequencies of CD85j^+^ and PD-1^+^ cells. In addition, compared to PBNK from ccRCC patients, TINK exhibited higher expression of activation markers, tissue residency features and decreased expression of the activating receptors DNAM-1, NKp30, NKp46, NKp80 and CD16, suggesting a more inhibitory phenotype. Analysis of The Cancer Genome Atlas (TCGA) revealed that CD48, CD45, CD85j and PD-1 are significantly overexpressed in ccRCC and that their expression is associated with an NK cell infiltration signature. Calculation of z-scores revealed that their expression on PBNK, alone or combined, distinguished ccRCC patients from HD. Therefore, these molecules emerge as novel potential biomarkers and our results suggest that they might constitute possible targets for immunotherapy in ccRCC patients.

## Introduction

Renal cell carcinomas (RCC) constitute a malignancy of the kidney that, according to GLOBOCAN (https://gco.iarc.fr/), exhibited an incidence of 4.5 cases per 100,000 individuals in 2018. The most frequent RCC is clear cell RCC (ccRCC, 70-75% of all RCC), followed by papillary RCC and chromophobe RCC (cancer.gov). RCC patients diagnosed early have a good prognosis (81% of five-year survival for stage I tumors and 74% of five-year survival for stage II tumors). However, patients diagnosed at advanced stages have a dramatic drop in five-year survival (53% of five-year survival for stage III tumors and 8% of five-year survival for stage IV tumors). RCC patients can be treated by partial or radical nephrectomy or kinase inhibitors. Also, immunotherapy with checkpoint inhibitors that target the PD-1/PD-L1 axis recently emerged as a treatment option in advanced disease (1, 2). However, most of them are diagnosed at later stages and exhibit recurrence and metastases, without further therapeutic options (3, 4). Therefore, it is of major interest to discover novel targets for immunotherapy and to identify biomarkers that might indicate recurrence or the efficacy of a certain treatment. Eradication of tumor cells is mainly executed by NK cells and cytotoxic CD8^+^ T lymphocytes (CTL) through direct cytotoxicity against tumor cells and secretion of IFN-γ and other proinflammatory cytokines (5, 6). NK cells recognize specific ligands expressed on tumor cells that promote the engagement of an array of activating receptors such as DNAM-1, NKG2D, 2B4, NKG2C, the Natural Cytotoxicity Receptors (NCR) NKp30, NKp44, NKp46 and NKp80, and others (5). However, NK cell activation is counterbalanced by the engagement of an array of inhibitory receptors such as CD85j (ILT2), KIR, NKG2A, and TIGIT, and NK cell activity is also regulated by coinhibitory molecules such as PD-1 (5, 7).

Human PBNK comprise 2 major subsets, according to the expression of CD56 (8). Approximately 90% of PBNK exhibit a CD3^-^CD56^dim^ phenotype and a robust cytotoxic activity. The other 5-10% of PBNK display a CD3^-^CD56^bright^ phenotype, are abundant in secondary lymph nodes, display poor cytotoxic activity and mainly produce IFN-γ and other cytokines in response to different stimuli (8–10). Activation of CD56^dim^ NK cells induces several changes that include the up-regulation of the expression of CD25 and CD69, and the downregulation of the expression of CD62L and CCR7 (8, 11–13). Moreover, during activation of NK cells, downregulation of CD62L and CCR7 prevents their egress from the tissue and migration into lymph nodes, and therefore their downregulation is considered a feature of tissue residency (8, 11, 12). In addition, upregulation of CD69, traditionally associated with activation, has been recently associated with tissue residency features (8, 11–13). Also, acquisition of CD57 expression by human NK cells has been associated with terminal differentiation (8, 13–15).

The mechanism of action of NK cells in the control of human hematologic malignancies is relatively well understood (16, 17). However, their role in the control of solid tumors is less known. Although NK cells and CTL infiltrate RCC tumors (18), tumors manage to grow and metastasize mainly due to the existence of an immune suppressive tumor microenvironment (TME). Through local and systemic effects, TME may generate dysfunctional NK cells with an altered phenotype, and the detection of such abnormal phenotypic characteristics of PBNK and TINK may result in the identification of novel targets for immunotherapy and/or biomarkers.

It was observed that TINK from ccRCC patients exhibit a diverse expression of KIR, CD85j and NKG2A (19), and that the extent of NK cell infiltration and the expression of CD16 and lytic mediators is associated with the functional capacity of these TINK (19, 20). Reduced expression of NKp46 was also described in TINK from ccRCC patients (21). Also, it has been observed that a lower frequency of TINK express the activating receptors NKp46, NKG2D, NKG2C, and a higher frequency of TINK express the inhibitory receptors NKG2A, CD158a and CD158b (22). Moreover, TINK from ccRCC display impaired degranulation (21, 22) and cytokine production (22). Others also described an abnormal phenotype of PBNK in RCC patients (84% of which were ccRCC) such as an increased expression of PD-1 that correlated with disease stage and was significantly reduced after surgical removal of the tumor (23). Phenotypic alterations of NK cells might be different in PBNK compared to TINK because TINK establish a close interaction with the TME while PBNK are only exposed to systemic effects of the TME. However, the comparative characteristics of PBNK and TINK from ccRCC remain mostly ill-defined. Such studies may unravel phenotypic alterations of NK cells that may represent useful prognostic, therapeutic and follow-up biomarkers and/or lead to the discovery of new candidates for immunotherapy, especially considering that immunotherapies aimed at reinvigorating NK cells in RCC constitute promising approaches (24).

Therefore, the aim of this work was to perform a phenotypic analysis of previously poorly explored markers expressed by PBNK and TINK from ccRCC patients to elucidate if systemic and local effects affect these cells critically involved in the elimination of tumor cells. We complemented these studies with bioinformatic analyses of TCGA data to establish whether the overexpression of inhibitory receptors detected in this work on NK cells from ccRCC patients is a general characteristic of ccRCC tumor samples and is associated with a NK cell signature.

## Materials and Methods

### Patients and Samples

Peripheral blood mononuclear cells (PBMC) were isolated from blood of healthy donors (HD, provided by the Blood Bank of the Hospital Churruca-Visca of Buenos Aires) or kidney cancer patients (drawn just before nephrectomy) by Ficoll-Paque™ Plus (GE Life Sciences) centrifugation. Blood and nephrectomies were provided by the urology service from the Centro de Educación Médica e Investigaciones Clínicas “Norberto Quirno” (CEMIC) from the city of Buenos Aires. The characteristics of the patients are listed in **Table 1**. A total of 12 patients with ccRCC and 13 HD were included in the study. This study was conducted according to the guidelines of the Declaration of Helsinki, and approved by the Institutional Ethics Committee of IBYME (protocol CE003-03/2014, date of approval: March 20, 2014). Also, informed consent was obtained from all subjects involved in the study.

**Table 1:** Patients with ccRCC included in the study.

Patient	Gender	Age	Stage	Type of nephrectomy*
1	M	74	Fuhrman II	P
2	M	74	Fuhrman II	R
3	M	67	Furhman II (pT3bpN0)	R
4	M	61	Fuhrman II	P
5	M	63	Fuhrman II	P
6	M	67	Fuhrman II/III	R
7	F	59	Fuhrman II	R
8	M	59	Fuhrman II	R
9	M	74	Fuhrman II	P
10	M	73	Fuhrman IV	R
11	M	71	Fuhrman II	R
12	F	72	Fuhrman II	P

*P, partial; R, radical.

### Preparation of Tumor Cell Suspensions

Surgical biopsies of human RCC were obtained from patients subjected to partial or radical nephrectomy and used for the preparation of single cell suspensions. Briefly, tumors were cut into small pieces and subjected to mechanical dissociation and filtration through nylon mesh (0.45 µm) in the presence of phosphate-buffered saline. After washing with saline solution, immune cells were enriched by Ficoll-Paque™ Plus (GE Life Sciences) centrifugation. All procedures were performed on ice. Diagnosis of each RCC was confirmed by the pathology service and only data corresponding to ccRCC were presented in this study.

### Antibodies and Reagents

The following mAb were used for flow cytometry (FC): APC anti-CD56 (N901) from Beckman Coulter; PE/Cy7 anti-CD3 (UCHT1) from TONBO; FITC anti-DNAM-1 (DX11) from BD; FITC anti-CD85j (292305), AlexaFluor488 anti-TRAIL (71908), PE anti-NKG2A (131411) and AlexaFluor488 anti-NKG2C (134591) from Biotechne; FITC anti-CD16 (3G8); FITC anti-CD69 (FN50); PE anti-CD25 (BC96); PE anti-CD62L (DREG-56); PE anti-NKG2D (1D11), PE anti-NKp30 (P30-15); PE anti-NKp46 (9E2); PE anti-NKp80 (5D12); PE anti-2B4 (C1.7); FITC anti-CD48 (BJ40); APC/Cy7 anti-CD45 (HI30); AlexaFluor488 anti-PD-1 (29E.2A3); FITC anti-HLA class I (W6/32); PE anti-FasL (NOK-1); PE anti-NKp44 (9E2); FITC anti-CD57 (HCD57); FITC anti-CCR7 (G043H7); FITC anti-CD27 (M-T271); and PE anti-TIGIT (A15153G) from Biolegend.

### Flow Cytometry

FC was performed as described (25, 26). Non-specific staining was blocked with 10% normal mouse serum. Cells were analyzed in a FACSCanto II flow cytometer (BD). Data were analyzed using FlowJo X software (BD) and results were expressed as geometric mean fluorescence intensity (MFI), as relative MFI (rMFI) calculated as the MFI of the specific mAb divided by the MFI of the “fluorescence minus one” (FMO) control or as percentage of positive cells. For comparison of PBNK with TINK, we used rMFI because the FMO of the tumor samples was higher than the FMO of the blood samples.

### Bioinformatic Analysis

The Tumor IMmune Estimation Resource (TIMER, http://timer.cistrome.org/) (27) and the Gene Expression Profiling Interactive Analysis 2 (GEPIA2, http://gepia2.cancer-pku.cn/) (28) were used to analyze publicly available RNAseq data from The Cancer Genome Atlas (TCGA). TIMER was used to study the differential expression between ccRCC tumor and adjacent normal tissues datasets for the different genes of interest, and the statistical significance was computed by differential analysis (edgeR). GEPIA2 was used for correlation analysis between a multi-gene NK cell signature (*NCR1, XCL2, IL2RB, KLRF1, KIR2DL4, KLRC3, XCL1, NKG7, CTSW, NCR3* and *IL18RAP*) and the genes of interest (29, 30).

### Statistical Analysis

**Figure 1:**
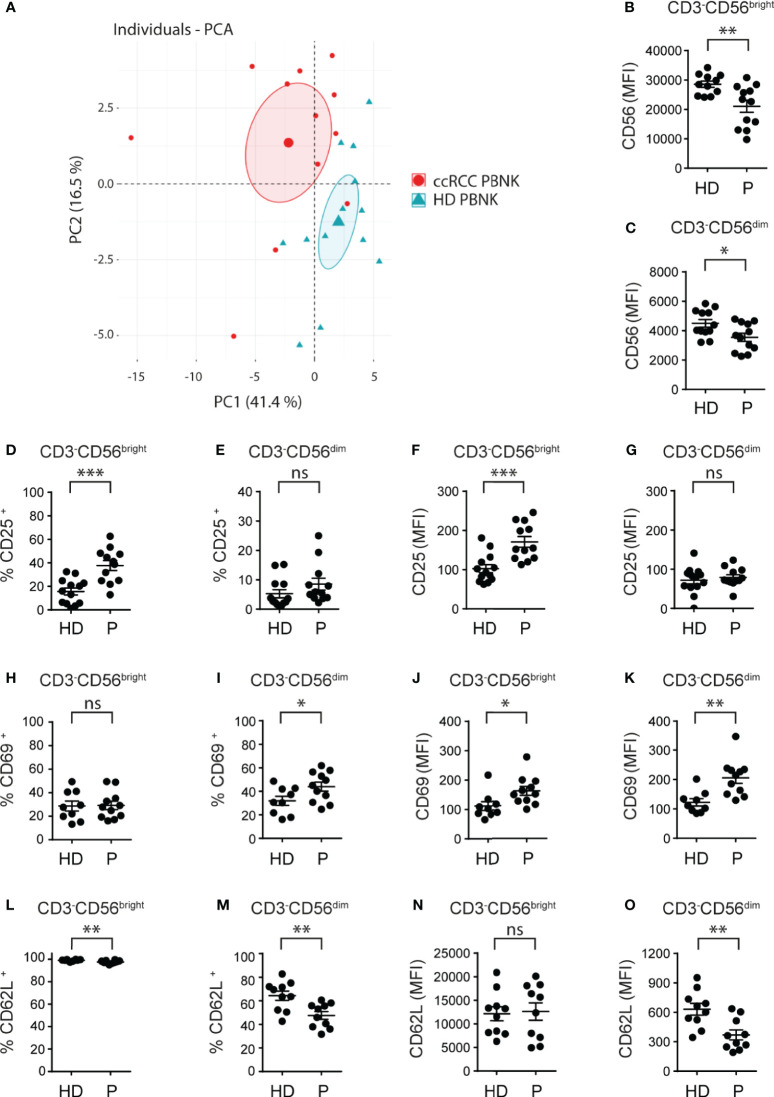
Cell surface markers differentiate ccRCC patients from HD and indicate that PBNK from ccRCC patients exhibit an activated phenotype. MFI of the molecules analyzed on CD3^-^CD56^bright^ and CD3^-^CD56^dim^ cells of HD and ccRCC PBNK were used to perform a PCA **(A)**. The graph of individuals with the confidence ellipse is shown. Also, PBNK cells from healthy donors (HD) and ccRCC patients (P) were analyzed by FC to compare the intensity of expression of CD56 on CD3^-^CD56^bright^ **(B)** and on CD3^-^CD56^dim^ NK cells **(C)**, the frequencies of CD25^+^ cells in CD3^-^CD56^bright^ **(D)** and in CD3^-^CD56^dim^ NK cells **(E)**, the intensity of expression of CD25 on CD3^-^CD56^bright^ **(F)** and on CD3^-^CD56^dim^ NK cells **(G)**, the frequencies of CD69^+^ cells in CD3^-^CD56^bright^ **(H)** and in CD3^-^CD56^dim^ NK cells **(I)**, the intensity of expression of CD69 on CD3^-^CD56^bright^ **(J)** and on CD3^-^CD56^dim^ NK cells **(K)**, the frequencies of CD62L^+^ cells in CD3^-^CD56^bright^ **(L)** and in CD3^-^CD56^dim^ NK cells **(M)**, and the intensity of expression of CD62L on CD3^-^CD56^bright^ **(N)** and on CD3^-^CD56^dim^ NK cells **(O)**. For HD: *n*=11 **(B)**, *n*=12 **(C)**; *n*=13 **(D–G)**; *n*=9 **(H–K)** and *n*=10 **(L–O)**. For P: *n*=12 **(B–G)**, *n*=11 **(H–K)**, and *n*=10 **(L–O)**. A two-sided unpaired t-test with Welch’s correction was used in **(B–D**, **F–I, K–O)**. A two-sided unpaired t-test with Mann-Whitney´s correction was used in **(E, J)**. ns, not significant; *p < 0.05; **p < 0.01; ***p < 0.001.

Principal component analysis (PCA) of scaled data was conducted in R (version 4.0.3) (31) with missMDA (32) to compute missing values. For the comparison of PBNK from HD with PBNK from ccRCC patients (**Figure 1A**), the MFI of CD56, CD62L, CCR7, CD16, CD25, CD69, FasL, TRAIL, CD48, CD27, CD45, MHC-I, PD-1, DNAM-1, NKG2D, NKp30, NKp44, NKp46, NKp80, NKG2C, 2B4, NKG2A, and TIGIT on CD56^bright^ and CD56^dim^ NK cells, and the MFI of CD85j and CD57 on CD56^dim^ cells were used. For the comparison of PBNK with TINK from ccRCC patients (**Figure 3A**), the rMFI of the following markers were used: CD62L, CCR7, CD16, CD25, CD69, FasL, TRAIL, CD48, CD27, CD57, MHC-I, PD-1, DNAM-1, NKG2D, NKp30, NKp44, NKp46, NKp80, NKG2C, 2B4, NKG2A, TIGIT, and CD85j. Plots were generated using the package factoextra (33). z-scores for CD85j, CD45 and CD48 expression were calculated using the formula z=(x-µ)/σ, where z is the “z-score”, x is the MFI of CD45 expression or the frequency of CD85j^+^ or CD48^+^ from total PBNK in each individual sample, µ is the mean of the MFI of CD45 expression or the mean of the frequency of CD85j^+^ or CD48^+^ PBNK cells in the HD population, and σ is the standard deviation of each set of these data in the HD population. The sum of z-scores was calculated for patients for whom data were available for each parameter (receptors). To compare PBNK from HD with PBNK from ccRCC patients, a two-sided unpaired t-test with Welch´s correction (when samples passed the normality test) or with Mann-Whitney´s correction (when samples did not pass the normality test) was used. To compare PBNK with TINK from ccRCC patients, a two-sided paired t-test was used when samples passed the normality test, and a two-sided paired t-test with Wilcoxon rank test was used when samples did not pass the normality test. D´Agostino-Pearson was used as normality test. Data were analyzed using Prism 6.0 software (GraphPad).

## Results

### PBNK From ccRCC Patients Exhibit an Activated Phenotype With an Altered Balance of Activating and Inhibitory Receptors

First, we phenotyped PBNK from ccRCC patients and HD (the characteristics of the patients are detailed in **Table 1**). NK cells were defined as CD3^-^CD56^+^ cells and NK cell subsets were further characterized as CD3^-^CD56^bright^ and CD3^-^CD56^dim^ cells using the gating strategy described in **Supplementary Figure 1**. A PCA of cell surface markers used to analyze NK cells phenotype demonstrated that, according to PC1 and PC2, PBNK from ccRCC patients segregated together and separated from HD (**Figure 1A**). Therefore, as PBNK from ccRCC patients and HD could be discriminated with these markers, we explored the expression of these receptors and molecules in more detail.

Compared to HD, ccRCC patients exhibited similar frequencies of PBNK and distribution of CD3^-^CD56^bright^ and CD3^-^CD56^dim^ cells (*not shown*) but with lower amounts of CD56 expression in both NK cell subsets (**Figures 1B, C**). Moreover, CD3^-^CD56^bright^ but not CD3^-^CD56^dim^ cells from ccRCC patients showed a significant increased frequency of CD25^+^ cells (**Figures 1D, E**) and they expressed significantly higher amounts of CD25 (**Figures 1F, G**). In addition, only CD3^-^CD56^dim^ cells but not CD3^-^CD56^bright^ cells from ccRCC patients displayed a significant increased frequency of CD69^+^ cells (**Figures 1H, I**), and both subsets from ccRCC patients expressed significantly higher amounts of CD69 (**Figures 1J, K**). Moreover, CD3^-^CD56^bright^ and CD3^-^CD56^dim^ NK cells from ccRCC patients presented a lower frequency of CD62L^+^ cells, a finding that was most notorious in CD3^-^CD56^dim^ cells (**Figures 1L, M**) and was accompanied by significantly lower expression of CD62L only on CD3^-^CD56^dim^ NK cells (**Figures 1N, O**). Also, we did not observe differences in the expression of CCR7, CD27 and CD57 on either subpopulation of PBNK from ccRCC patients compared to HD (*not shown*). Overall, our results show that PBNK from RCC patients display features of activated NK cells.

**Figure 2:**
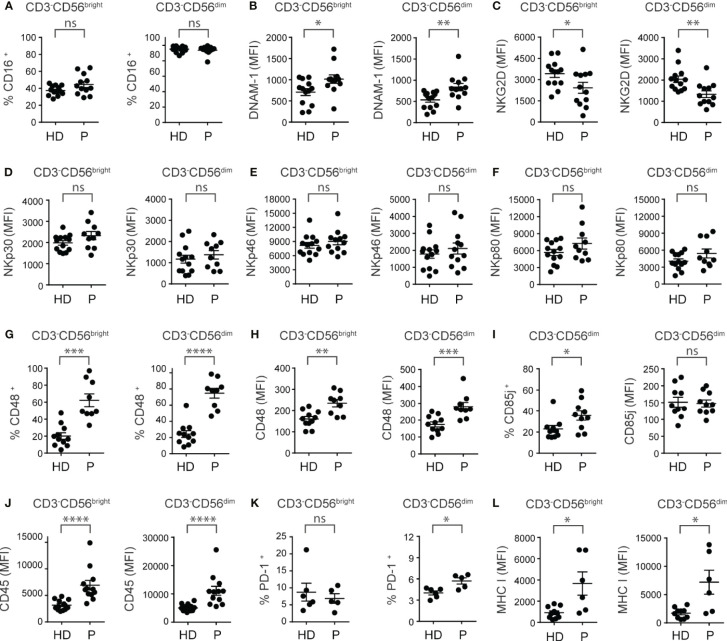
PBNK from ccRCC patients exhibit an altered phenotype characterized by a dysregulation in the expression of activating and inhibitory receptors. PBNK cells from healthy donors (HD) and ccRCC patients (P) were analyzed by FC to compare, in the CD3^-^CD56^bright^ (*left graph*) and CD3^-^CD56^dim^ cells (*right graph*) subsets, the frequency of cells that expressed CD16 **(A)**, the intensity of expression of DNAM-1 **(B)**, NKG2D **(C)**, NKp30 **(D)**, NKp46 **(E)**, NKp80 **(F)**, the frequency of cells that expressed CD48 **(G)**, and the intensity of expression of CD48 **(H)**. Also, we analyzed the frequency of CD85j^+^ cells (*left graph*) and the intensity of expression of CD85j (*right graph*) in CD3^-^CD56^dim^ cells **(I)**. In addition, in CD3^-^CD56^bright^ (*left graph*) and CD3^-^CD56^dim^ cells (*right graph*) we analyzed the intensity of expression of CD45 **(J)**, the frequency of PD-1^+^ cells **(K)**, and the intensity of expression of MHC-I **(L)**. For HD: *n*=13 **(A–F, J)**, *n*=11 **(G, H)**, *n*=10 **(I, L)** and *n*=6 **(K)**. For P: *n*=12 **(A–C**, **E, J)**, *n*=1 **(D, F, I)**, *n*=9 **(G, H)**, *n*=5 **(K)** and *n*=6 **(L)**. A two-sided unpaired t-test with Welch´s correction was used in left graphs of **(A–C**, **G, H)**, in both graphs of **(D–F)**, and in right graph of **(I)** A two-sided unpaired t-test with Mann-Whitney´s correction was used in right graphs of **(A–C, G, H)**, in left graph of **(I)** and in both graphs of **(J–L)**. ns, not significant; *p < 0.05; **p < 0.01; ***p < 0.001; ****p < 0.0001.

Next, we analyzed the expression of a set of activating and inhibitory receptors that regulate NK cell activity in CD3^-^CD56^bright^ and CD3^-^CD56^dim^ NK cells from ccRCC patients compared to HD. Among the activating receptors analyzed, we observed that there were no differences in the frequency of CD16^+^ NK cells (**Figure 2A**). However, we observed an increased expression of DNAM-1 (**Figure 2B**), a reduced expression of NKG2D (**Figure 2C**), and no changes in the expression of NKp30 (**Figure 2D**), NKp46 (**Figure 2E**), NKp80 (**Figure 2F**), NKG2C (*not shown*), 2B4 (*not shown*) and NKp44 (*not shown*) in both subsets of PBNK. We also detected a higher frequency of NK cells that expressed the 2B4 ligand CD48 (**Figure 2G**) in the CD3^-^CD56^bright^ and CD3^-^CD56^dim^ NK cell subsets from ccRCC patients compared to HD. Both subsets of PBNK from ccRCC also exhibited increased amounts of CD48 (**Figure 2H**). In addition, among the inhibitory receptors and other molecules involved in negative signaling, we did not observe differences in the expression of NKG2A and TIGIT in CD3^-^CD56^bright^ and CD3^-^CD56^dim^ NK cells from ccRCC patients compared to HD (*not shown*). Also, we observed that CD3^-^CD56^bright^ did not express CD85j *(not shown)* but that CD3^-^CD56^dim^ cells from ccRCC patients exhibited higher frequencies of CD85j^+^ cells but with similar amounts of expression compared to HD (**Figure 2I**). In addition, CD3^-^CD56^bright^ and CD3^-^CD56^dim^ NK cells from ccRCC patients exhibited a substantially higher expression of CD45 (**Figure 2J**), while only CD3^-^CD56^dim^ PBNK cells from ccRCC patients presented a higher frequency of PD-1^+^ cells (**Figure 2K**) without changes in the amount of PD-1 expression (*not shown*). We also observed that CD3^-^CD56^bright^ and CD3^-^CD56^dim^ NK cells from ccRCC patients displayed higher expression of MHC-I (**Figure 2L**).

In addition, we did not observe differences in the frequency and amounts of expression of molecules involved in the cytotoxic function of NK cells *via* the death receptor pathway, such as FasL and TRAIL, in both subsets of PBNK from ccRCC patients compared to HD (*not shown*).

In summary, compared to HD, PBNK from ccRCC patients exhibit features of activated NK cells with a dysbalanced array of activating and inhibitory cell surface receptors skewed towards an inhibitory phenotype.

### TINK Exhibit Activated and Tissue Residency Characteristics With a Balance of Activating and Inhibitory Receptors Even More Markedly Skewed Towards an Inhibitory Phenotype

**Figure 3:**
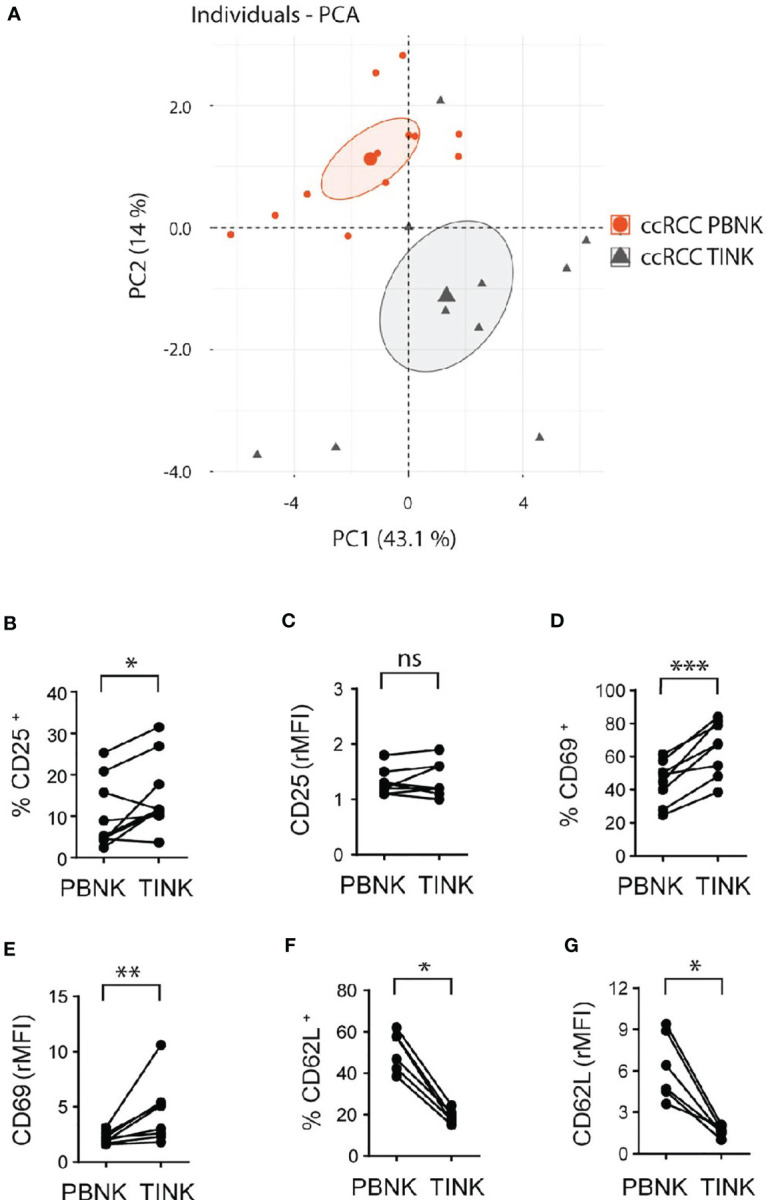
Cell surface markers differentiate TINK from PBNK in ccRCC and indicate that TINK from ccRCC patients exhibit an activated phenotype. rMFI of the molecules analyzed on CD3^-^CD56^+^ cells from PBNK and TINK from ccRCC patients were used to perform PCA **(A)**. The graph of individuals with the confidence ellipse is shown. Also, PBNK and TINK from ccRCC patients were analyzed by FC to compare the frequency of CD25^+^ cells **(B)**, the intensity of expression of CD25 **(C)**, the frequency of CD69^+^ cells **(D)**, the intensity of expression of CD69 **(E)**, the frequency of CD62L^+^ cells **(F)**, and the intensity of expression of CD62L **(G)**. In **(C, E, G)**, relative MFI (rMFI) was used instead of MFI because the FMO was different for PBNK and TINK. *n*=9 **(B, C)**; *n*=8 **(D, E)**; *n*=6 **(F, G)**. A two-sided paired t-test was used in **(B, D)** A two-sided paired t-test with Wilcoxon rank test was used in **(C, E–G)**. ns, not significant; *p < 0.05; **p < 0.01; ***p < 0.001.

To analyze the features of TINK and considering that CD56^bright^ and CD56^dim^ NK cell subsets could not be assessed separately within the tumor, we used the gating strategy described in **Supplementary Figure 2**. A PCA of the same cell surface markers used to analyze PBNK demonstrated that, according to PC1 and PC2, PBNK from ccRCC patients could be differentiated from TINK (**Figure 3A**). Hence, as with PBNK, we explored these differences in more detail.

Compared to PBNK from paired ccRCC patients, TINK exhibited similar amounts of expression of CD56 (*not shown*), and a higher frequency of CD25^+^ (**Figure 3B**) with similar expression of CD25 (**Figure 3C**). TINK also displayed a higher frequency of CD69^+^ cells (**Figure 3D**) and expressed higher amounts of CD69 (**Figure 3E**). In addition, they showed a lower frequency of CD62L^+^ cells (**Figure 3F**) and expressed less CD62L (**Figure 3G**) than PBNK. Also, we did not find differences in the expression of CCR7, CD27 and CD57 (*not shown*). Thus, TINK from ccRCC patients also display features of activated NK cells with tissue residency characteristics.

**Figure 4:**
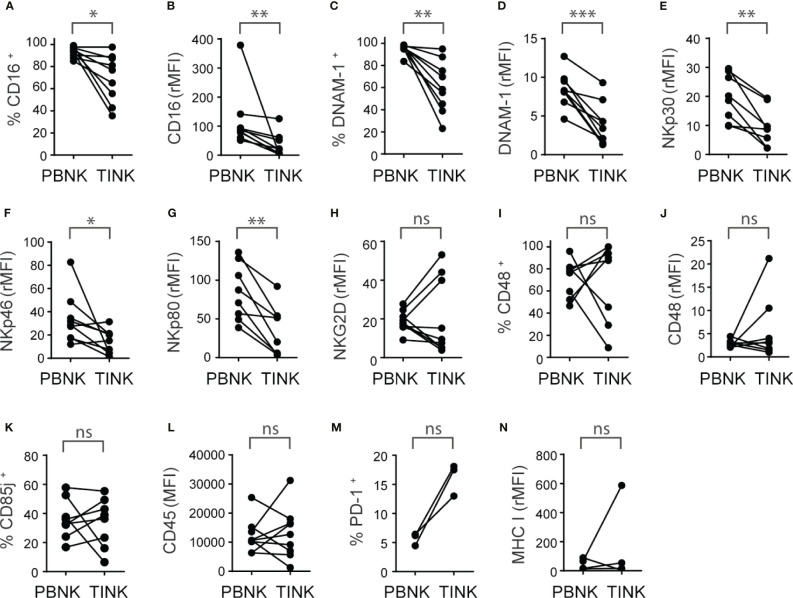
TINK from ccRCC patients exhibit a similar increased expression of inhibitory receptors than PBNK and additional decreased expression of activating receptors. Peripheral blood NK cells (PBNK) and tumor-infiltrating NK cells (TINK) from ccRCC patients were analyzed by FC to compare the frequency of CD16^+^ cells **(A)**, the intensity of expression of CD16 **(B)**, the frequency of DNAM-1^+^ cells **(C)**, the intensity of expression of DNAM-1 **(D)**, the intensities of expression of NKp30 **(E)**, NKp46 **(F)**, NKp80 **(G)** and NKG2D **(H)**, the frequency of CD48^+^ cells **(I)**, the intensity of expression of CD48 **(J)**, the frequency of CD85j^+^ cells **(K)**, the intensity of expression of CD45 **(L)**, the frequency of PD-1^+^ cells **(M)** and the intensity of expression of MHC-I **(N)**. In **(B, D–H, J, N**), the relative MFI (rMFI) was used instead of MFI because the FMO was different for PBNK and TINK. *n*=9 **(A–D**, **F, H, L)**; *n*=8 **(E, G, I–K)**; *n*=3 **(M)**; *n*=4 **(N)**. A two-sided paired t-test was used in **(A, D, E, G–I)** and **(K)** A two-sided paired t-test with Wilcoxon rank test was used in **(B, C, F, J, L, N)**. ns, not significant; *p < 0.05; **p < 0.01; ***p < 0.001.

The analysis of the expression of activating receptors revealed that TINK, compared to PBNK from ccRCC patients, exhibited a reduced frequency of CD16^+^ cells (**Figure 4A**) and expressed less CD16 (**Figure 4B**). Similarly, TINK presented a reduced frequency of DNAM-1^+^ cells (**Figure 4C**) and expressed less DNAM-1 (**Figure 4D**). TINK also exhibited reduced expression of NKp30 (**Figure 4E**), NKp46 (**Figure 4F**) and NKp80 (**Figure 4G**), but similar expression of NKG2D (**Figure 4H**) and frequencies and intensity of expression of NKp44 and NKG2C (*not shown*). Also, we did not observe differences in the frequency of CD48^+^ NK cells (**Figure 4I**) or in the expression of CD48 (**Figure 4J**). In addition, the analysis of the expression of some inhibitory receptors demonstrated that there were no differences in the frequency of CD85j^+^ (**Figure 4K**), NKG2A^+^ and TIGIT^+^ cells (*not shown*) or in the amount of these three molecules (*not shown*) or CD45 (**Figure 4L**) expressed by PBNK and TINK. Furthermore, there was a trend towards a higher frequency of PD-1^+^ cells in TINK compared to PBNK that did not reach statistical significance (**Figure 4M**), while the amount of expression of MHC-I (**Figure 4N**) was similar in both NK cell compartments.

In addition, we observed no changes in the frequency of FasL^+^ and TRAIL^+^ TINK and in the intensity of expression of FasL and TRAIL compared to PBNK (*not shown*).

In summary, TINK exhibit features of activated NK cells with tissue residency characteristics and a pattern of activating and inhibitory receptors that is skewed towards an even more inhibitory phenotype than PBNK from ccRCC patients.

### Bioinformatic Analyses Confirm That Overexpression of CD85j, CD45, CD48 and PD-1 in TINK Is Associated With a NK Cell Tumor Infiltration Signature in ccRCC

**Figure 5:**
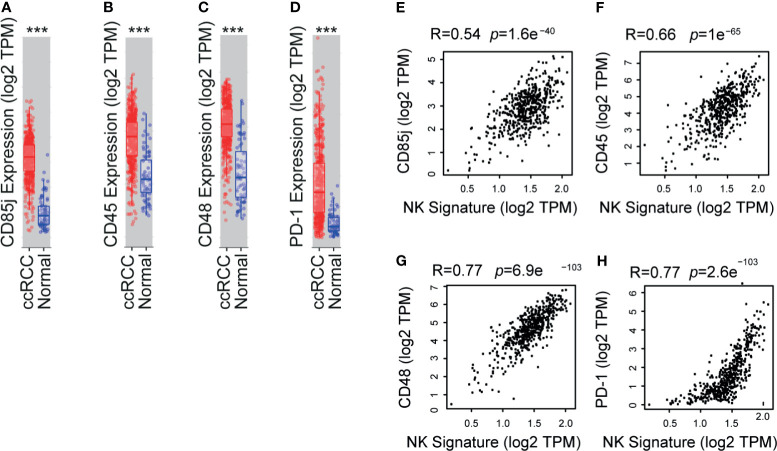
TCGA analyses shows that CD85j, CD45, CD48 and PD-1 are overexpressed in ccRCC and associated with a NK cell signature. Analysis of expression of CD85j **(A)**, CD45 **(B)**, CD48 **(C)** and PD-1 **(D)** in ccRCC (*n*=533) compared to normal kidney (*n*=72) using the TIMER platform with data deposited in the TCGA. Also, a correlation analysis between the expression of CD85j **(E)**, CD45 **(F)**, CD48 **(G)** and PD-1 **(H)** and a NK cell signature (established by the overexpression of a group of 11 NK cell-associated genes that are not expressed or are weakly expressed on CD8^+^ T cells: NCR1, XCL2, IL2RB, KLRF1, KIR2DL4, KLRC3, XCL1, NKG7, CTSW, NCR3, and IL18RAP) in ccRCC was performed using the GEPIA2 platform. ***p < 0.001.

To interrogate whether the abnormal expression of NK cell receptors observed in our study is a general feature of patients with ccRCC, we performed a bioinformatic analysis using the TCGA database. We assessed whether inhibitory molecules that exhibited overexpression in PBNK and TINK compared to HD *a)* were encoded by genes that exhibited altered expression in ccRCC compared to healthy kidney, and *b)* were associated with a NK cell infiltration signature in ccRCC samples. This signature was established by the overexpression of a group of 11 NK cell-associated genes that are not expressed or are weakly expressed on CD8^+^ T cells: *NCR1, XCL2, IL2RB, KLRF1, KIR2DL4, KLRC3, XCL1, NKG7, CTSW, NCR3*, and *IL18RAP*. We observed that CD85j (**Figure 5A**), CD45 (**Figure 5B**), CD48 (**Figure 5C**) and PD-1 (**Figure 5D**) were significantly overexpressed in ccRCC. Moreover, expression of CD85j (**Figure 5E**), CD45 (**Figure 5F**), CD48 (**Figure 5G**) and PD-1 (**Figure 5H**) were strongly associated with a NK cell infiltration signature. Therefore, our results suggest that higher expression of CD45 on NK cells, increased frequencies of CD85j^+^ and higher expression and frequency of CD48^+^ NK cells (as detected by multicolor FC) might be a common characteristic of patients with ccRCC. Moreover, as their expression distinguishes ccRCC patients from HD, they also might emerge as potential candidates for immunotherapy.

### Calculation of z-Scores for CD85j, CD45 and CD48 Unravel Their Potential Use as Biomarkers

**Figure 6:**
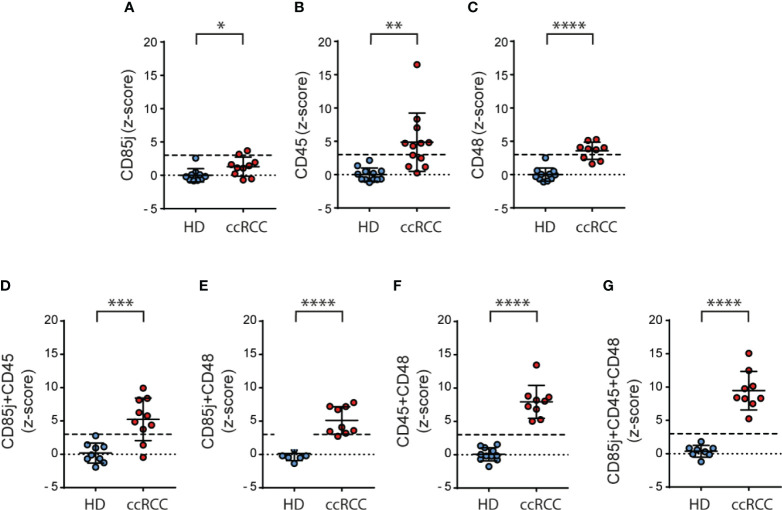
Normalization of the expression of CD85j, CD45 and CD48 on NK cells significantly discriminates between ccRCC and HD samples. Normalization of the expression of CD85j, CD45 and CD48 in PBNK was performed calculating the respective z-scores and individual z-scores for CD85j **(A)**, CD45 **(B)** and CD48 **(C)** were depicted. Also, the sum of z-scores was calculated for the pairs of receptors CD85j and CD45 **(D)**, CD85j and CD48 **(E)**, and CD45 and CD48 **(F)** as well as the sum of z-scores of CD85j, CD45 and CD48 **(G)**. The dashed line in each graph indicates the value of the mean plus 3 SD. For HD: *n*=10 **(A)**, *n*=12 **(B)**, *n*=11 **(C, F)**, *n*=9 **(D)** and *n*=8 **(E, G)**. For P: *n*=10 **(A, D)**, *n*=12 **(B)** and *n*=9 **(C, E–G)**. *p < 0.05; **p < 0.01; ***p < 0.001; ****p < 0.0001.

To explore if these inhibitory receptors could be part of a signature that might be used as biomarkers, we normalized our results calculating the z-scores for CD85j, CD45 and CD48, and we also combined these z-scores to explore if the behavior of PBNK from ccRCC differs from HD. We observed that z-scores from CD85j (**Figure 6A**), CD45 (**Figure 6B**) and CD48 (**Figure 6C**) in PBNK were significantly higher in ccRCC patients than in HD. Furthermore, the sum of z-scores by pairs of receptors improved the discrimination power between ccRCC patients and HD (**Figures 6D–F**). However, the most remarkable discrimination power was achieved with the sum of z-scores from the three receptors, as the mean for ccRCC patients was 9.5 standard deviations above the mean of HD and every single patient’s value was at least 5 standard deviations above the mean of the HD (**Figure 6G**).

In summary, besides PD-1, which is an already known target for immunotherapy, our results suggest that expression of CD85j, CD45 and CD48 on NK cells from ccRCC patients constitute potential biomarkers and they might constitute candidates for therapeutic intervention that deserve further investigation.

## Discussion

Most patients with advanced ccRCC experience tumor recurrence and metastases (34). Notably, ccRCC is a tumor that does not exhibit a high mutational burden (35) and therefore this finding might explain tumor progression even in the presence of high NK cell and CD8 T cell infiltration (36). However, patients with RCC treated with anti-PD-1 or anti-PD-L1 mAb respond relatively well and exhibit a higher objective response than the mean trend (37). Therefore, an immunosuppressive TME plays an important role in the generation of dysfunctional cytotoxic cells. Importantly, even though the administration of the immunological checkpoint inhibitors nivolumab and ipilimumab, alone or combined with other therapies, opened new therapeutic opportunities (38), most of the patients still do not benefit from these treatments. Therefore, the identification and validation of novel targets in immuno-oncology that, alone or combined with PD-1 blockade, may reinvigorate the function of cytotoxic cells becomes crucial but constitutes a formidable challenge. Bioinformatic analysis based mainly on RNA expression in whole tumor samples does not allow for discrimination of the different cells that constitute the TME. Additionally, such analysis does not account for post-translational modifications that may impact on cell surface expression of candidate molecules. Therefore, the analysis of the coding RNA may not mirror the protein expression. Consequently, the analysis of peripheral blood cells and paired tumor-infiltrating cells becomes a valuable tool in the path of identification and validation of novel predictive biomarkers and targets in immuno-oncology (39).

Given the relevant role of NK cells during tumor immunity and the promising pipelines in the field of NK cell therapies (40), we performed an exhaustive phenotypic analysis of NK cells from ccRCC patients with the aim to select candidates for further investigation of their potential use as biomarkers and/or targets for immunotherapy.

Interestingly, the panel of markers used in this work enabled us to discriminate ccRCC PBNK from HD, as shown by PCA. A more detailed analysis of the differences between HD and ccRCC PBNK phenotype revealed that compared to HD, PBNK from ccRCC patients exhibited features of activated NK cells shown by the increased frequency and expression of CD25 and CD69, and a decreased expression of CD62L (8, 41, 42). These PBNK did not exhibit alteration in their maturation or terminal differentiation status (according to the expression of CD27 and CD57, respectively) but displayed an abnormal array of activating and inhibitory receptors. The most notable change in the activating receptors was the decreased expression of NKG2D on PBNK from ccRCC patients. NKG2D is critical for tumor elimination (43, 44) and its down-regulation may arise as a consequence of the presence of soluble ligands such as MICA in plasma of cancer patients, a fact that contributes to tumor progression and immune escape (45, 46). Also, although there were no differences in the expression of the activating receptor 2B4, PBNK from ccRCC patients displayed increased frequencies and expression of its ligand CD48 (47). Notably, *cis* interaction between 2B4 and CD48 on NK cells has been reported to reduce the availability of 2B4 to interact with CD48 in *trans* on tumor cells, resulting in a heightened NK cell activation threshold (48). In addition, under certain circumstances 2B4-CD48 engagement leads to functional inhibition of NK cells (49). Moreover, CD48 can also bind to CD2 and this interaction and the effect of CD48 engagement on the cells that express it has not been properly studied yet. Therefore, up-regulation of CD48 on PBNK from ccRCC may contribute to tumor resistance to NK cell-mediated effector functions.

Other remarkable changes in PBNK from ccRCC patients were the increased frequencies of CD3^-^CD56^dim^ cells that expressed the inhibitory receptor CD85j and the coinhibitory molecule PD-1, and the expression of higher amounts of CD45 and MHC-I on both subsets of NK cells. CD85j engagement on NK cells limits their effector functions (26, 50) and its up-regulation in PBNK from triple negative breast cancer patients has been associated with impaired antibody-dependent cell-mediated cytotoxicity (ADCC) elicited by Cetuximab (51). HLA-G, which binds to CD85j with higher affinity than the classical MHC-I (35), is expressed in RCC cells (36, 37) and plays an inhibitory role on NK cell-mediated cytotoxicity against tumor cells (37). CD45 is a receptor with tyrosine phosphatase activity that participates in fine-tuning of cellular responses (52–54). Its phosphatase activity negatively controls the activation threshold of different cells from the immune system to diverse stimuli and alteration in such signaling has been involved in different pathological conditions. Regarding the upregulation of MHC-I observed on NK cells, such effect also may contribute to their inhibition as it was shown that cross-linking of MHC-I molecules on human NK cells inhibits their function (55, 56), while protecting them from self-killing (fratricide) through engagement of 2B4 (57). MHC-I expression on tumors cells might also play an important role in preventing NK cells activation through engagement of inhibitory receptors. Therefore, PBNK from ccRCC patients, besides exhibiting an activated status exhibited a phenotype skewed towards inhibition or higher activation threshold and could be clearly distinguished from PBNK from HD with the surface molecules analyzed in this work.

Moreover, performing a PCA we also demonstrated that PBNK from ccRCC patients differ from TINK. A more detailed phenotypic analysis revealed that TINK also exhibited an activated phenotype with tissue residency features characterized by a higher frequency of CD25^+^ and of CD69^+^ cells, higher expression of this last molecule, lower frequency of CD62L^+^ and DNAM-1^+^ cells and lower expression of both molecules. Also, TINK did not exhibit alteration in their maturation or terminal differentiation status but exhibited a reduced frequency of CD16^+^ cells and diminished expression of CD16, NKp30, NKp46 and NKp80, but conserved the reduced expression of NKG2D detected in PBNK. Such phenotype may not only impair direct tumor recognition by TINK through many activating receptors but also may weaken their ability to trigger ADCC through CD16. Regarding inhibitory receptors, TINK maintained the increased frequency of CD85j^+^ and CD48^+^ cells observed in PBNK from ccRCC patients. Moreover, each of the 3 patients analyzed for PD-1 expression exhibited increased frequency of PD-1^+^ cells in TINK compared to PBNK, but such increase was not significant likely due to the low number of samples analyzed. TINK also exhibited similar heightened expression of CD45 and MHC-I as PBNK from ccRCC. Overall, our results indicate that TINK exhibit an activated phenotype with tissue residency characteristics that is even more skewed towards inhibition than PBNK from ccRCC patients. Coopting regulatory circuits that result in the upregulation of inhibitory receptors such as CD85j, CD45, CD48 and PD-1 may raise NK cell activation threshold, resulting in hyporesponsive NK cells. Such tumor-driven subversion of NK cells may impose restrictions to their effector functions to control tumor progression and metastases. In addition, within the TME, a concomitant downregulation of activating receptors also would desensitize NK cells to tumor cells that express the specific ligand, further facilitating tumor progression.

In addition, a bioinformatic analysis revealed that, compared to healthy kidneys, increased expression of CD85j, CD45, CD48 and PD-1 is a general characteristic of ccRCC and is strongly associated with an NK cell tumor infiltration signature in this type of tumor. Thus, besides the well-known role of PD-1 blockade on cytotoxic CD8^+^ T cells, our results suggest that NK cells might also be involved in the efficacy of anti-PD-1/PD-L1 immunotherapy in ccRCC. Also, CD85j, CD45 and CD48 emerge as novel potential targets whose inhibition or blockade should be further examined as it may promote the reinvigoration of TINK. In addition, to evaluate the potential of these molecules as a signature capable of differentiating patients and HD, we calculated z-scores for each receptor and used them alone or combined. Remarkably, the sum of z-scores for CD85j, CD45 and CD48 exhibited a strong discrimination ability between ccRCC and HD samples. Therefore, we postulate their potential utility as peripheral biomarkers in ccRCC.

In summary, PBNK from ccRCC patients display an inhibitory profile characterized by overexpression of CD85j, CD45, CD48 and PD-1, while TINK exhibit additional alterations characterized by a decreased expression of several activating receptors. Therefore, our results suggest that CD85j, CD45 and CD48 expression represent interesting potential biomarkers in ccRCC and they might constitute possible candidates for immunotherapy whose blockade may result in their validation as novel targets in immuno-oncology.

## Data Availability Statement

The raw data supporting the conclusions of this article will be made available by the authors, without undue reservation.

## Ethics Statement

The studies involving human participants were reviewed and approved by Institutional Ethics Committee of IBYME. The patients/participants provided their written informed consent to participate in this study.

## Author Contributions

AZ performed and designed most of the experiments and analyzed the data. XI, SN, NT, FS, JS, RS, MF, and CD contributed experimentally to the data presented in some figures. XI also performed the PCA analysis. AR and FS provided the nephrectomies and the data of the patients. NZ conceived, designed, and supervised the study and wrote the manuscript. All the authors reviewed the manuscript. All authors contributed to the article and approved the submitted version.

## Funding

This work was funded with grants from the National Agency for Promotion of Science and Technology from Argentina (ANPCYT), the National Research Council of Argentina (CONICET) and the Trust in Science Program from GlaxoSmithKline (GSK), all to NZ. We also thank Fundación Williams and Fundación René Barón for providing financial assistance (donations) to our laboratory.

## Conflict of Interest

The authors declare that the research was conducted in the absence of any commercial or financial relationships that could be construed as a potential conflict of interest.
