# Supplementary material for: Circulating and Tumor-Infiltrating NK Cells From Clear Cell Renal Cell Carcinoma Patients Exhibit a Predominantly Inhibitory Phenotype Characterized by Overexpression of CD85j, CD45, CD48 and PD-1
Source: Front Immunol. 2021 Jun 4;12:681615. doi: 10.3389/fimmu.2021.681615 (PMC8212993; doi:10.3389/fimmu.2021.681615)
Supplement: Supplementary file 2 [file DataSheet_2.pdf]

Supplementary Figure 2.

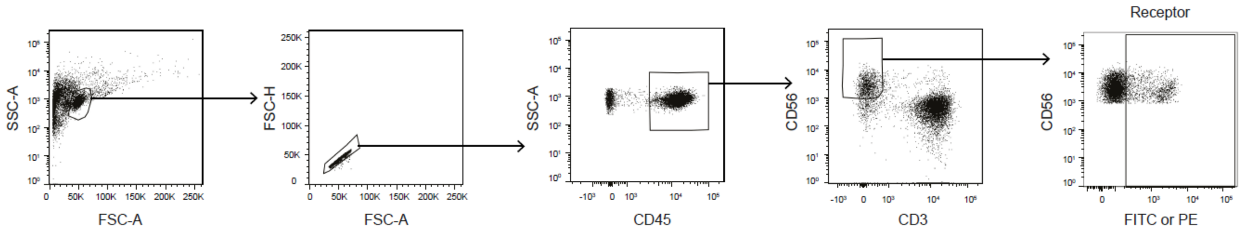

Supplementary Figure 2. Strategy used to gate TINK from ccRCC patients, and to assess the expression of cell surface receptors. NK cells were defined as $CD3^{-}CD56^{+}$ cells in the $CD45^{+}$ cell population.
